# 

**Published:** 1862-06

**Authors:** 


TERRE-HAUTE, Ind,, June 18, 1862.

The "Indiana State Dental Association" will hold its eighth semi-annual session in the City of Indianapolis, commencing on the *second Tuesday of July*, prox., at 2 o'clock, P. M.

It is particularly desired that every member of the Association will be present. And the entire profession, and physicians, both in and out of the State, are most cordially invited to attend this meeting, and coöperate with the Association to further the cause of dental progress.

By order of the President, P. G. C. HUNT.

S. R. SMITH, *Rec. and Cor. Sec'y.*

---

BUFFALO, N. Y., May 10th, 1862.

THE AMERICAN DENTAL CONVENTION will hold its Eighth Annual Session at Trenton Falls, N. Y., commencing on Tuesday, the fifth day of August next, at 10 o'clock A.M. You are respectfully and cordially invited to attend.

The Executive Committee reported, at the last Annual Convention, the following order of business, and subjects for discussion, for the next Convention :

ORDER OF BUSINESS.

1st. Admission of Members.
2d. Reading Minutes of the last Convention.
3d. Report of Officers and Committees.
4th. Election of Officers.
5th. Retiring President's Address.
6th. Induction of Officers.

All essays shall be read to open the discussion on the subjects to which they relate.

No member shall speak more than ten minutes, nor more than twice on the same subject without permission.

I. *Miscellaneous Subjects.*—1. Anæsthetics. Their use and relative value. 2. Alveolar abscess. 3. The causes influencing an abnormal development of the teeth.

II. *Operative Dentistry.*—1. Filling Teeth. Simple and complicated cavities. 2. The Dental pulp. Its varied treatment. 3. The extraction of teeth.

III. *Mechanical Dentistry.*—1. Artificial Dentures. Temporary and Permanent.

IV. *Unfinished Business.*

N. B.—The Executive Committee suggest that half an hour every morning be devoted to the presentation of models, improvements and inventions, and the disposal of business not embodied in the regular order.

Signed,

W. H. Atkinson,

G. T. Barker,

W. B. Roberts,

I. J. Wetherbee,

Samuel Mallet,

} *Executive Committee.*

This is a Convention where all regular practicing Dentists may unite in the free discussion of subjects relating to, and the full interchange of opinions, experience and practice of the Science of Dentistry. As associated action is the surest means for advancement, it is hoped that by personal attendance, the contribution of essays, and discussion of the subjects which have been presented by the Executive Committee, you will add to the interest of the Convention, and the elevation and advancement of the profession of Dental Surgery.

Yours, Very Respectfully,
B. T. WHITNEY.

---

The American Dental Association will meet in the City of Cleveland, Ohio, upon the last Tuesday of July next, at ten o'clock A. M.

W. Muir Rogers,

*Corresponding Sec'y.*

*Shelbyville, Ky.*,

May 26, 1862.

Committees are to report at that meeting upon the following subjects, viz.:

1. *Dental Physiology.*
2. *Dental Pathology and Surgery.*
3. *Mechanical Dentistry.*
4. *Dental Education.*
5. *Dental Literature.*
6. *Dental Chemistry.*

Upon each of these we expect full and interesting reports will be presented. Ed.

### CONTENTS.

### DENTAL REGISTER,

### OF THE WEST.

### Issued Monthly, at $3 a year in advance.

DEVOTED TO THE INTERESTS OF THE DENTAL PROFESSION.

**Edited by J. TAFT and GEO. WATT.**

The Volume commences in January and closes in December.

The Register being issued monthly is always fresh, therefore indispensable to the practitioner who desires to keep posted up with the new inventions and improvements which are daily developed. Neither expense nor effort will be spared to give value and variety to its contents. Articles will be illustrated with well executed engravings, whenever they will add to the interest or assist in explanation.

Its extensive circulation and frequency of issue makes it the BEST DENTAL ADVERTISING MEDIUM in the United States.

☞CONTRIBUTIONS SOLICITED.

Address Original Communications to **Geo. Watt, Xenia, O.**

Exchanges to **J. Taft**, or **Dental Register, Cincinnati, Ohio.**

Business Communications to

J. TAFT, Publisher,
56 West Fourth Street, Cincinnati, O.

JNO. T. TOLAND, Proprietor,
38 West Fourth Street, Cincinnati, O.

Communications must reach the Editor as early as the 15th, to insure insertion in the next issue.

